# Adherence of Patients With Breast Carcinoma and Gynecologic Genital Carcinoma in the Use of Integrative Therapy Recommendations

**DOI:** 10.1177/15347354261483630

**Published:** 2026-09-15

**Authors:** Milena Beierlein, Carolin C. Hack, Anna-Katharin Theuser, Katharina Au, Lena Brueckner, Matthias W. Beckmann, Katharina Seitz

**Affiliations:** 1 Department of Gynecology and Obstetrics, Erlangen University Hospital, Comprehensive Cancer Center Erlangen–European Metropolitan Area Nuremberg (CCC ER-EMN), 9171 Friedrich Alexander University of Erlangen–Nuremberg, Erlangen, Germany; 2 27168 Institute for Women’s Health/Institut Fuer Frauengesundheit GmbH, Erlangen, Germany

**Keywords:** integrative medicine, adherence, complementary and alternative medicine, gynecology, oncology

## Abstract

**Background:**

This study aimed to evaluate differences in adherence to integrative therapy recommendations provided by the Consultation Service for Integrative Medicine (SIM) at the Women’s Clinic of the University Hospital Erlangen, Germany, across patient subgroups. The study sought to identify opportunities for further optimization and refinement of the integrative care program.

**Methods:**

In this cross-sectional study, data of 120 women who visited the SIM consultancy were collected at the first presentation (Baseline) and after 3 to 6 months of therapy (Follow-Up) using standardized questionnaires. The adherence variable was defined as the proportion of integrative therapy methods implemented for at least four weeks of the total number of therapy recommendations received per patient. Differences in patient-reported adherence to integrative therapies among cohorts defined by demographic characteristics, lifestyle factors, and tumor-related characteristics were evaluated using independent-samples t-tests and analyses of variance (ANOVA).

**Results:**

There was no significant difference in adherence with Integrative therapy recommendations in cohorts based on age, education, Body-mass-index (BMI), tumor localization and treatment modality. Factors significantly associated with lower adherence, were low physical activity, defined as self-reported absence of regular exercise, compared with patients reporting approximately 1 hour or 2–4 hours of exercise per week, and presence of distant metastases.

**Conclusion:**

Adherence with SIM recommendations was significantly lower among patients with low physical activity levels, as well as among patients with distant metastatic disease. This shows that the treatment concepts of the SIM need to be adapted more individually and, in particular, body-based treatments need to be reviewed in order to make them accessible for patients with poorer physical condition.

## Introduction

The use of complementary and alternative medicine (CAM) plays a considerable role, particularly among patients with cancer. A 2012 meta-analysis reported the percentage of cancer patients using CAM at around 40%. Furthermore an increase in prevalence has been recorded over the last decades, particularly after the year 2000.^1^

When looking at gynecological cancer patients an even higher prevalence has been demonstrated in numerous studies. A review on the use of complementary and alternative medicine (CAM) in gynecological oncology describes a prevalence of about 70% in women with breast cancer between 2002 and 2006, with the lowest value being described as 30% and the maximum as high as 90%.^1^ Slightly lower percentages were found for patients with gynecologic carcinomas compared to breast cancer patients. The values given ranged from the middle 40th percentile to 76% as a maximum.^1-3^ These high rates of application show the need to address CAM in professional patient care.

Most frequently used methods are biologically-based treatments, for example herbs, teas and vitamin or nutritional supplements. Furthermore, manipulative or body-based methods are popular, such as sports, chiropractic therapy and massage. These are often complemented by relaxation techniques, spiritual therapies or applications of homeopathy, anthroposophic or naturopathic treatment and traditional Chinese medicine (TCM).^3-6^

Even though data on many CAM methods are heterogeneous and quantitative data on efficacy often is lacking, general benefits and positive effects on quality of life, reduction of side effects and better coping with cancer have been demonstrated.^7-12^

However, the aim should not be to only provide complementary medicine, but to develop integrative medical care. Bell^13^ defines Integrative Medicine as a higher-ranking care system that pursues holistic well-being as its primary goal. The bio-psycho-socio-spiritual dimensions are taken into account, combining both conventional and CAM approaches. The decisive factor in demarcation and definition of integrative medicine is that CAM methods are not a substitute for conventional medicine but are integrated into conventional therapy. This means the integration of conventional medicine and complementary methods into a common patient care, common guidelines and common values and goals for a holistic treatment of the patient.^14,15^

To implement this in clinical practice the Consultation Service for Integrative Medicine (SIM), a specialized outpatient clinic was established. Based on current symptoms, ongoing treatments, and individual goals, a personalized integrative treatment plan is developed to safely integrate complementary approaches into conventional gynecological care. It does not offer alternative treatments as a substitute for standard care, but rather focuses on supportive strategies.

The program includes safety-validated methods from classical naturopathy, such as physical activity, phytotherapy, nutritional counselling, relaxation techniques, and hydrotherapy, as well as adjunctive interventions including micronutrients, probiotics, vitamins, trace elements, immunomodulatory or enzyme-based therapies, alongside general lifestyle recommendations.

In this context the consultation concept of the SIM at the Erlangen Women’s Clinic was evaluated and is to be further optimized as an example of structured, safe and effective holistic care. Practicability of complementary and integrative medicine (CIM) shall be assessed by analyzing the patients’ adherence on integrative therapy recommendations and potential influencing factors on implementation rates.

## Methods

### Study Design and Patients

The present study is a cross-sectional study, conducted between November 2016 and February 2021 at the Women’s Clinic of the University Hospital Erlangen.

Study participants were recruited prospectively in the SIM. The general inclusion criteria were a minimum age of 18 years, female gender and breast cancer or gynecological cancer. Patients who did not speak German and patients who had problems understanding the content of the study or the questionnaires were excluded from the study. A written declaration of consent was provided by all study participants.

Data was initially collected at the first visit to the consultation using a standardized paper-based questionnaire, internally named “Integrative Medicine (IMed) basic questionnaire”, which was completed independently by the patients. It was developed specifically for SIM consultancy and validated in terms of comprehensibility, complexity, scope and patient satisfaction.^16^ The questionnaire contained information on general social factors, lifestyle, gynecological history and risk factors as well as concomitant diseases. This was followed by questions about tumor disease, therapies and current symptoms. The survey also asked about any previous experience with integrative methods and treatment goals regarding integrative medicine.

Detailed individual and interdisciplinary treatment recommendations were developed based on the questionnaire, anamnesis, and physical examination during the consultation. The integrative methods were adjusted to current treatments to avoid interactions and to effectively address complaints and side effects.

The individual therapy concept was comprehensively explained during the second personal consultation and handed out in written form. Afterwards patients were responsible for implementing the treatment suggestions themselves.^16^

The follow-up data were collected three to six months later in order to ensure a sufficient time interval for implementation and effect of integrative therapies. For this purpose a second paper-based, standardized “IMed follow-up questionnaire” was developed. The questions were systematically addressed to the patient by a member of the research team in a personal contact during one of the appointments at the clinic or by telephone.^7^ Individual details of the basic questionnaire, initial consultation and treatment plan for each patient were used to follow up on complaints, treatment goals and implementation of recommendations. To evaluate the consultation, satisfaction with the overall and therapy concept, its practicability and comprehensibility as well as satisfaction with the atmosphere and organization of the consultation were surveyed. The follow-up also included questions on new complaints, any new therapy goals, monthly costs and suggestions for improvement.

Data of the basic questionnaire, the follow-up surveys and data of the hospital’s own patient files on tumor disease and therapy were taken into account in the evaluations.

The study protocol was approved on September 13, 2016 by the Ethics Committee of the Friedrich-Alexander-University Erlangen/Nuremberg with the reference number 255_16 B.

### Statistical Analysis

The data collected with the questionnaires and additional patient-specific information on tumor disease and therapy were recorded in a database. Statistical analysis was performed with SPSS Statistics for Windows version 28 (IBM Corporation, Armonk, New York, USA). Ambiguous and missing data, as well as the answer “no information”, were classified as “missing values” and not included in the analyses.

To analyze the therapy concept, mean, standard deviation, maximum and minimum values of the number of given recommendations and implementation rate per therapy recommendation was calculated.

In order to identify possible factors influencing implementation, the value of “adherence” was introduced, which describes the implementation rate per patient, calculated as the proportion of therapy recommendations consistently implemented out of the total number of recommendations received. An implementation period of at least 4 weeks was assessed as a consistently implemented recommendation.

Since a normal distribution could be assumed using Kolmogorov-Smirnov and Shapiro-Wilk test, independent t-tests and analyses of variance (ANOVA) were used to compare adherence mean values of different subpopulations. Bonferroni post-hoc tests performed case of significant differences. Influence of demographic characteristics (age group, health insurance status, level of education), lifestyle factors (BMI, smoking, physical activity) and tumor disease characteristics (tumor location, distant metastases and treatment modality) on implementation rates of integrative therapy recommendations was analyzed. Bonferroni correction was applied to adjust for multiple testing, resulting in a corrected significance level of α = 0.0056 (0.05/9).

## Results

**Figure 1.**
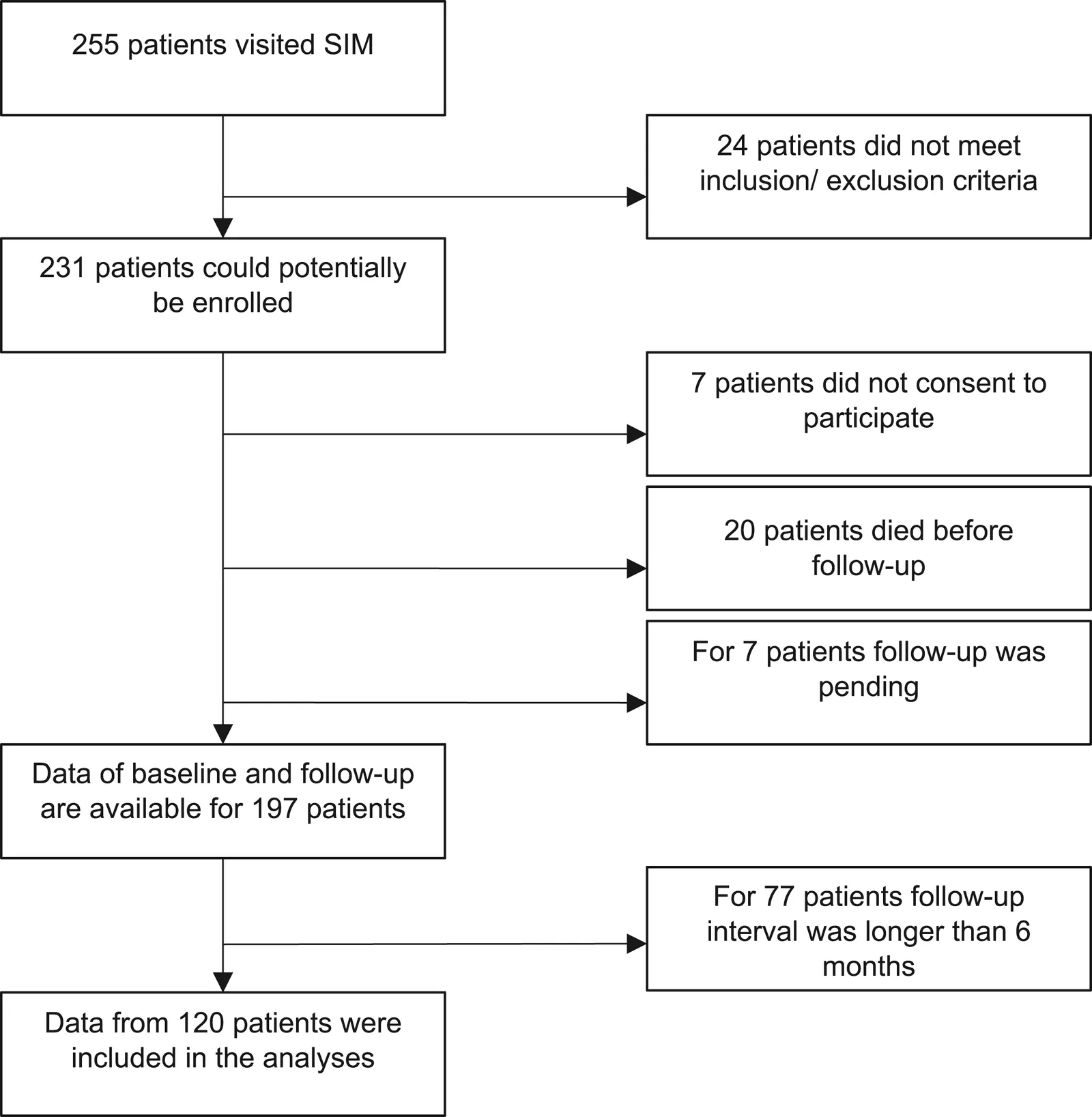
Composition of the patient collective

From establishment of the SIM in 2014 until November 2020, 255 patients visited the special consultation. Twenty-four patients did not meet the described inclusion and exclusion criteria, seven patients declined to participate in the study. Twenty women died before the follow-up survey, and for seven patients the follow-up was pending at the time of evaluation. Thus, data from both the baseline questionnaire and the follow-up survey, were available for 197 patients. In order to maintain comparability, a maximum interval of 6 months (with a tolerance of 2 additional weeks for postponements due to patient’s state of health or treatment) between the receipt of the treatment plan and the follow-up survey was defined for this evaluation. This led to the exclusion of a further 77 patients. In total, data of 120 women treated in the outpatient clinic for Integrative Medicine in the Women’s Hospital of the University Hospital Erlangen were analyzed (Figure 1).

Based on the patients’ complaints and treatment goals, an individual treatment plan was developed, which contained an average of 25 ± 6 recommendations. The recommended integrative therapy methods were basically structured according to the five pillars of Kneipp’s natural healing methods.^17^ These include nutritional therapy, which is taken up in the therapy concept through recommendations for a balanced, healthy diet. In addition, the regulative therapy deals with, for example, a structured daily routine or the use of relaxation exercises. Methods recommended in the field of exercise therapy included regular walking, defined daily step targets, moderate endurance or strength training. Examples of hydrotherapy applications are relaxation baths, cold facial casts, alternating casts or cold water applications to the extremities. Finally, the pillar of phytotherapy was taken up with therapy suggestions such as psyllium husks, lavender or the enzyme preparation Equinovo®. Other applications with sufficient evidence or experience outside the five pillars of naturopathy were also recommended. Table 1 summarizes the 40 most frequently recommended integrative methods and their respective implementation rate.

**Table 1.** Most Frequently Provided Treatment Recommendations in the SIM and Implementation of Each Recommendation (Number of Patients n = 120). for Implementation Rates Only Patients Who had Received the Recommendation in the Treatment Plan Were Considered

Therapy recommendation	Patients who received the recommendation n (%)	Patients who implemented the recommendation n (%)
Nutritional recommendations	118 (98 %)	92 (78 %)
Structured daily routine	118 (98 %)	106 (90 %)
Relaxation exercises	117 (98 %)	78 (67 %)
Regular walking/step goal	113 (94 %)	98 (87 %)
Cold facial pour in the morning	113 (94 %)	48 (42 %)
Spending time outdoors	106 (88 %)	94 (89 %)
Endurance training	105 (88 %)	56 (53 %)
Equinovo®	105 (88 %)	80 (76 %)
IMed-Infusions	104 (87 %)	64 (62 %)
Oil pulling	100 (83 %)	34 (34 %)
Consciously planning something nice once per week	94 (78 %)	80 (85 %)
Vitamin B Complex	90 (75 %)	82 (91 %)
Vitamin D	89 (74 %)	74 (83 %)
Light muscle training	84 (70 %)	38 (45 %)
Stimuli on hands and feet	82 (68 %)	68 (83 %)
Psycho-oncological Support	76 (63 %)	26 (34 %)
Tea mixture for liver and kidney	73 (61 %)	55 (75 %)
Peeling with olive oil and sugar	67 (56 %)	21 (31 %)
Mindfulness-based stress reduction	66 (55 %)	23 (35 %)
Cold water applications/alternating casts	60 (50 %)	25 (42 %)
Sleeping hygiene	60 (50 %)	40 (67 %)
Relaxation baths	53 (44 %)	12 (23 %)
Electromyostimulation training	47 (39 %)	8 (17 %)
Psyllium husks	46 (38 %)	18 (39 %)
Lavender	39 (33 %)	11 (28 %)
Ginger	38 (32 %)	29 (76 %)
Skin care (with urea)	37 (31 %)	30 (81 %)
Maintaining social contacts	35 (29 %)	33 (94 %)
Diarroesan®	25 (21 %)	7 (28 %)

To further examine the potential applications of integrative medicine, the “adherence parameter” was calculated for each patient. This was defined as the proportion of therapy recommendations implemented for at least four weeks in relation to the therapy recommendations received per patient. Adherence averaged across all study participants was 60.2 ± 14.4%. The lowest implementation rate was 22.0%. One patient was able to apply 100.00% of the treatment recommendations.

A central aim of the studies was to determine factors influencing the implementation and adherence of integrative therapies to better adapt them to individual patients. Firstly, the influence of demographic factors was examined. These were not significant for age groups (p = 0.776), health insurance (p = 0,012) and education (p = 0,982).

The calculated values for testing the demographic factors can be found in Table 2.

**Table 2.** Influence of the Demographic Factors Age Group, Health Insurance and Education on Patient Adherence With Treatment Recommendations of the SIM (Number of Patients n = 120). Missing Values Were Not Included

Influencing factor	Number of patients n	Mean of adherence	*p*
**Age group**			
≤40 years	19	61.9 %	0.776^a^
41 – 60 years	77	60.3 %	
≥61 years	24	58.7 %	
**Health insurance** ^c^			
Public health insurance	81	58.0 %	0.012^b^
Private/supplementary health insurance	37	65.2 %	
**Education** ^d^			
Secondary school	44	60.5 %	0.982^a^
High school diploma (Abitur)	19	61.3 %	
University degree	53	60.6 %	

^a^ANOVA.

^b^Independent sample t-Test Significance level α = 0.0056.

^c^Missing values: 2.

^d^Missing values: 4.

Further on the possible influence of lifestyle factors on adherence was analyzed. The analysis of variance did not reveal any significant difference in adherence between the subgroups for the BMI category (p = 0.145) and smoking behavior (p = 0.796). With regard to physical activity, however, ANOVA showed significantly different values (p < 0.001). Bonferroni post-hoc test revealed a significant difference in adherence between patients who exercised “2-4 hours per week” or “approx. 1 hour per week” compared to patients who stated that they “never” exercised. The results on the influence of lifestyle factors on adherence are summarized in Table 3.

**Table 3.** Influence of Lifestyle Factors: BMI Category, Alcohol Consumption, Smoking Behavior, Physical Activity, Low-Fat Diet and Consumption of Fruit and Vegetables on Patient Adherence With the SIM Treatment Recommendations (Number of Patients n = 120). Missing Values Were Not Included

Influencing factor	Number of patients n	Mean of adherence	*p*
**BMI** ^b^			
Underweight (BMI < 18,5)	7	56.3 %	0.145^a^
Normal weight (BMI 18,5 - 24,9)	66	60.6 %	
Overweight (BMI 25 - 29,9)	30	64.8 %	
Obesity (BMI ≥ 30)	12	54.6 %	
**Do you smoke or have you smoked?** ^c^			
No, never	71	60.5 %	0.796^a^
Yes, in the past	38	59.6 %	
Yes, i currently smoke	10	63.1 %	
**Do you exercise on a regular basis?** ^d^			
Yes, more than 4 hours per week	12	57.7 %	<0.001^a^
Yes, about 2-4 hours per week	42	64.9 %	
Yes, about 1 hour per week	31	63.6 %	
No, never	25	51.0 %	

^a^ANOVA Significance level α = 0.0056.

^b^Missing values: 5.

^c^Missing values: 1.

^d^Missing values: 10.

Tumor location and the presence of distant metastases were examined as possible disease-related influencing factors. Whether the carcinoma was localized in the breast or in the genitals did not show a significant difference in the respective adherence (p = 0.202). For patients without distant metastases, adherence was higher than for patients with distant metastases, with a marginally significant p-value of p = 0.005. When analyzing the influence of the treatment modality at the first visit to the SIM, no significant difference was demonstrated (p = 0.497). Table 4 shows the results of the tests for disease-related influencing factors.

**Table 4.** Influence of Disease Factors: Tumor Location, Distant Metastases and Therapy at the First Visit to the SIM on Patient Adherence With the SIM’s Therapy Recommendations (Number of Patients n = 120). Missing Values Were Not Included

Influencing factor	Number of patients n	Mean of adherence	*p*
**Localisation of primary disease** ^c^			
Mamma	91	61.1 %	0.202^b^
Genital	28	57.1 %	
**Distant metastases**			
No	93	62.2 %	0.005^b^
Yes	27	53.5 %	
**Therapy at first visit of the SIM**			
Follow-up	2	66.7 %	0.497^a^
Local therapy	15	63.6 %	
Systemic therapy	103	59.6 %	

^a^ANOVA.

^b^Independent sample t-Test Significance level α = 0.0056.

^c^The patient with concomitant breast and ovarian cancer was not included in this analysis.

## Discussion

At SIM, each patient receives an individual therapy concept adapted to her treatment, complaints and wishes. This includes recommendations from all five pillars of classic natural healing methods based on Sebastian Kneipp. Other therapies in the field of complementary medicine were also recommended, such as enzyme or micronutrient therapies, for which there is sufficient evidence and experience.^16-18^

Almost all patients (98 % in each case) received advice on nutrition and regulatory therapy, for example a structured daily routine and relaxation techniques. In most cases, recommendations for regular exercise, sporting activity and spending time outdoors were part of the concept. Depending on the symptoms, recommended preparations and phytotherapeutics often included enzyme therapies (e.g. bromelain, papain and lectin), vitamin infusions, vitamin D, vitamin B, tea blends, lavender, psyllium husks and ginger. Other important and frequent components of therapy concepts were supportive measures for mental and emotional well-being, such as psycho-oncological care, integrating relaxation breaks, consciously planning something enjoyable, Mindfulness-Based Stress Reduction or sleep hygiene. In the context of hydrotherapy, cold facial casts, cold water applications of all kinds and relaxation baths were recommended. Some therapy suggestions were more specifically adapted to individual complaints, so that these were less frequently recommended overall.

As part of the follow-up survey, the implementation of individual therapy components by the study participants was evaluated. Recommendations were considered to have been implemented if they were used for at least four weeks.

Recommendations that could be easily integrated into everyday life, such as a structured daily routine, regular exercise, skin care (with urea), consciously planning enjoyable events and maintaining social contacts, were implemented in more than 80%. Vitamin D and vitamin B were also taken in 83 % and 91 % of cases respectively. This is consistent with other studies conducted in Germany, in which the use of vitamins, minerals, micronutrients and exercise therapy were among the most common CIM applications, too.^19,20^ Enzyme preparations of bromelain, papain and lectin and ginger were used by 76 % of patients for at least four weeks. Adherence with other recommended preparations and phytotherapeutics varied in the analyses. Study participants who were already receiving a large number of medications as part of conventional therapies (oncologicals, supportive therapy, concomitant medications for comorbidities) might not want to take any more additional preparations. The high costs of some therapies could also be a possible negative factor.^21^

The conversion rates were lower for specific, for example oil pulling, olive oil-sugar peeling or the application of a hot and humid hay bag were only used in 24-34% of cases over a four-week period. Possible reasons might be lacking personal conviction, time required or the burden of other therapies. It must be seen as a limitation that the patients could have been asked what hurdles they saw in the implementation process themselves.

In addition to analyzing the implementation rates of single recommendations, potential factors influencing patients’ general adherence with integrative medicine were investigated. For this purpose, the adherence value, describing the proportion of therapy recommendations implemented in relation to all therapy recommendations received, was calculated for each patient. On average, a concept comprised 25 ± 6 therapy suggestions, of which an average of 60 ± 14.4 % per patient were implemented for at least four weeks. In order to improve therapy concepts, possible factors influencing adherence with integrative medicine should be identified. The implementation rate calculated in the study can serve as a parameter to analyze which patients can implement the concept successfully and thus benefit from the integrative methods. On the other hand, recommendations for patient collectives with lower adherence can be optimized to better meet individual needs and possibilities. As adherence was assessed by patients’ subjective verbal reports in the follow-up survey, a possible bias must be acknowledged.

First, adherence was evaluated in terms of demographic aspects. No significant difference was found between different age groups, public versus private health insurance or between levels of education considered. Existing studies have shown that patients with a high level of health awareness and physical activity are more likely to use CAM methods.^22-24^ Considering the difference in adherence levels depending on lifestyle factors, no significant influence of BMI and smoking behavior was found in this analysis.

In contrast, patients whose answer to the question about regular exercise was “no, never” showed significantly lower adherence in implementing the therapy concept than women who stated that they exercised regularly (2-4 h/week or 1 h/week) on their first visit to the SIM.

The therapy recommendations for exercise and sport include, for example, regular walking with a step target, moderate endurance training of approximately 2.5 hours per week, strength training or EMS (electro-myo-stimulation) training. It can be assumed that patients who have already been regularly active in sports are better able to do so because of their existing routine and motivation than patients who have never been active in sports. Existing sporting activity indicates a higher level of health awareness, a better general physical condition and greater discipline, so that patients may be more motivated and generally better able to make further efforts to carry out integrative therapies.^24^

As potential influencing factors of the tumor disease on adherence, the localization of the disease (breast versus genital carcinoma), the presence of distant metastases and the different treatment modalities were considered. There was a marginally significant difference between patients with and without distant metastases. Adherence was lower among study participants with organ or skeletal metastases than among women without distant metastases.

In order to ensure a holistic and comprehensive treatment, the recommendations of the SIM are usually very comprehensive. Implementing them requires a great deal of organization, energy and time. Particularly in patients with distant metastases, the patient’s limited state of health may be a reason why integrative methods recommended in the SIM cannot always be implemented. In these cases, the treatment concept needs to be individually adapted to the patient’s disease situation.

## Conclusion

In summary, many integrative recommendations were well implemented into everyday life by the patients. In particular, general recommendations such as structured daily routine, maintaining social contacts, spending time outdoors, as well as regular walking or step goals and putting stimuli on hands and feet against peripheral neuropathy showed high adoption rates. The intake of vitamins and the consideration of general nutritional recommendations were also implemented widely.

Nevertheless, there are differences in adherence regarding the CIM methods. Implementation rates were significantly lower for less physically active study participants and women with distant metastases. Further research is needed to identify the exact reasons. Potential starting points for optimizing therapy concepts include motivating and supporting women who are less physically active and adapting the therapy concept to the general condition and individual possibilities, especially in the presence of distant metastases.
